# The role of the Mediterranean diet in reducing the risk of cognitive impairement, dementia, and Alzheimer’s disease: a meta-analysis

**DOI:** 10.1007/s11357-024-01488-3

**Published:** 2025-01-11

**Authors:** Mónika Fekete, Péter Varga, Zoltan Ungvari, János Tibor Fekete, Annamaria Buda, Ágnes Szappanos, Andrea Lehoczki, Noémi Mózes, Giuseppe Grosso, Justyna Godos, Otilia Menyhart, Gyöngyi Munkácsy, Stefano Tarantini, Andriy Yabluchanskiy, Anna Ungvari, Balázs Győrffy

**Affiliations:** 1 Institute of Preventive Medicine and Public Health, Semmelweis University, Budapest, Hungary; 2 Doctoral College, Health Sciences Program, Semmelweis University, Budapest, Hungary; 3 Vascular Cognitive Impairment, Neurodegeneration and Healthy Brain Aging Program, Department of Neurosurgery, University of Oklahoma Health Sciences Center, Oklahoma City, OK USA; 4 Oklahoma Center for Geroscience and Healthy Brain Aging, University of Oklahoma Health Sciences Center, Oklahoma City, OK USA; 5 Department of Health Promotion Sciences, College of Public Health, University of Oklahoma Health Sciences Center, Oklahoma City, OK USA; 6 International Training Program in Geroscience, Doctoral College/Institute of Preventive Medicine and Public Health, Semmelweis University, Budapest, Hungary; 7 Dept. of Bioinformatics, Semmelweis University, 1094 Budapest, Hungary; 8 Cancer Biomarker Research Group, Institute of Molecular Life Sciences, HUN-REN Research Centre for Natural Sciences, 1117 Budapest, Hungary; 9 Department of Vascular and Endovascular Surgery, Heart and Vascular Center, Semmelweis University, Budapest, Hungary; 10 Department of Rheumatology and Clinical Immunology, Semmelweis University, Budapest, Hungary; 11 Department of Biomedical and Biotechnological Sciences, University of Catania, Catania, Italy; 12 Center for Human Nutrition and Mediterranean Foods (NUTREA), University of Catania, Catania, Italy; 13 Dept. of Biophysics, Medical School, University of Pecs, 7624 Pecs, Hungary

**Keywords:** Pharmacology, Cognitive decline, Neurodegenerative diseases, Nutritional epidemiology, Prevention

## Abstract

Age-related cognitive impairment and dementia pose a significant global health, social, and economic challenge. While Alzheimer’s disease (AD) has historically been viewed as the leading cause of dementia, recent evidence reveals the considerable impact of vascular cognitive impairment and dementia (VCID), which now accounts for nearly half of all dementia cases. The Mediterranean diet—characterized by high consumption of fruits, vegetables, whole grains, fish, and olive oil—has been widely recognized for its cardiovascular benefits and may also reduce the risk of cognitive decline and dementia. To investigate the protective effects of the Mediterranean diet on cognitive health, we conducted a systematic literature review using PubMed, Web of Science, and Google Scholar, focusing on studies published between 2000 and 2024. The studies included in the meta-nalysis examined the adherence to the Mediterranean diet and the incidence of dementia and AD. We applied a random-effects model to calculate pooled hazard ratios (HRs) with 95% confidence intervals (CIs) and assessed heterogeneity through *I*-square statistics. Forest plots, funnel plots, and *Z*-score plots were used to visualize study outcomes. Of the 324 full-text records reviewed, 23 studies met the inclusion criteria. The combined HR for cognitive impairment among those adhering to the Mediterranean diet was 0.82 (95% CI 0.75–0.89); for dementia, the HR was 0.89 (95% CI 0.83–0.95); and for AD, the HR was 0.70 (95% CI 0.60–0.82), indicating substantial protective effects. Significant heterogeneity was observed across studies, though *Z*-score plots suggested sufficient sample sizes to support reliable conclusions for each condition. In conclusion, this meta-analysis confirms that adherence to the Mediterranean diet is associated with an 11–30% reduction in the risk of age-related cognitive disorders, including cognitive impairment, dementia, and AD. These findings underscore the Mediterranean diet’s potential as a central element in neuroprotective public health strategies to mitigate the global impact of cognitive decline and dementia and to promote healthier cognitive aging.

## Introduction

Age-related cognitive impairment and dementia represent a global health, social, and economic crisis [1]. With the elderly population rapidly increasing in the Western world, the number of individuals affected by cognitive decline is expected to double by 2030 and triple by 2050 [1–4]. Dementia is not a singular disease; it encompasses a range of conditions [5], all characterized by progressive cognitive impairment, often accompanied by functional and behavioral decline.

While Alzheimer’s disease (AD) has long been considered the leading cause of dementia, emerging evidence highlights the equally significant contribution of vascular cognitive impairment and dementia (VCID) [6–8], which accounts for nearly half of all dementia cases. Many patients present with mixed etiology dementia, where both AD and vascular components coexist. Recent research also reveals that AD itself has a substantial microvascular component, emphasizing the critical role of vascular health in both conditions [9–14]. Microvascular pathologies, including endothelial dysfunction, neurovascular impairment [11,15], cerebromicrovascular rarefaction, and related declines in cerebral blood flow, blood–brain barrier (BBB) disruption [13,14], and cerebral microhemorrhages, are key contributors to the pathogenesis of both VCID and AD. Despite this, there is a gap in knowledge regarding how lifestyle and dietary interventions that support vascular health can mitigate the progression of these diseases. Vascular risk factors like hypertension [16,17], obesity [18–24], diabetes [25,26], and hyperlipidemia are common to both VCID and AD, suggesting that interventions aimed at improving vascular health could confer protective effects against both conditions. In this context, the Mediterranean diet, widely recognized for its cardiovascular benefits [27–34] and stroke prevention [35–55], has garnered attention for its potential to reduce the risk of cognitive decline and dementia through synergistic vasoprotective and neuroprotective effects [56]. This diet, rich in fruits, vegetables, whole grains, legumes, olive oil, and fish, with low consumption of red meat and saturated fats [57], is increasingly being linked to improved cognitive outcomes. However, one critical area that confounds the current literature is the variation in the composition of the “Mediterranean diet” across different countries. The traditional Mediterranean diet is not a single, uniform concept but rather a diverse set of dietary patterns that differ considerably across various Mediterranean countries. These regional differences in food selection and preparation could contribute to the heterogeneity observed in studies assessing the impact of the Mediterranean diet on dementia risk. Moreover, other aspects of the Mediterranean lifestyle, such as higher levels of physical activity, social engagement, and cultural habits, may synergistically enhance the diet’s protective effects, making it challenging to isolate the specific role of the diet in dementia prevention. These lifestyle factors, although integral to the Mediterranean way of life, also exhibit significant regional variation, further complicating the ability to draw definitive conclusions about the standalone effects of the Mediterranean diet on cognitive health.

Given these complexities, the aim of this study was to perform a comprehensive meta-analysis that consolidates data from a wide variety of studies to better understand the relationship between adherence to the Mediterranean diet and the risk of cognitive impairment, dementia, and Alzheimer’s disease. By systematically analyzing these studies, the meta-analysis seeks to offer robust evidence on the Mediterranean diet’s effectiveness in preventing cognitive decline and dementia, while accounting for potential sources of heterogeneity and confounding factors.

## Methods

### Literature search

A comprehensive literature search was conducted for this meta-analysis across PubMed, Web of Science, and Google Scholar databases. The search was restricted to studies published between 2000 and 2024 to include the most recent and relevant studies. The listed keywords and their combinations were used in the search strategy (Table 1). No language restrictions were applied during the search, and full-text publications were considered. Additionally, the reference lists of the identified articles and related metaanalyses were reviewed to gather data and to locate further relevant studies.

**Table 1:** List of keyword combinations for research on the relationship between Mediterranean diet adherence and dementia, cognifive decline, or Alzheimer’s disease

Keywords
“Mediterranean diet”AND “dementia”
“Mediterranean diet” AND “Alzheimer's disease”
“Mediterranean diet” AND “cognitive decline”
“Mediterranean diet” AND “cognitive impairment”
“Mediterranean diet” AND “memory loss”
“Dietary patterns” AND “dementia”
“Dietary patterns” AND “Alzheimer's disease”
“Mediterranean diet adherence” AND “dementia”
“Mediterranean diet adherence” AND “Alzheimer's disease”
“Mediterranean diet adherence” AND “cognitive decline”

### Inclusion and exclusion criteria

The inclusion and exclusion criteria were established based on the following guidelines: population (P), exposure (E), comparison (C), outcome (O), and study design (S). The inclusion and exclusion criteria were applied by two independent reviewers (AL, MF) who assessed the studies identified through the literature search. Studies that met the following criteria were included in the metaanalysis (Table 2). The aim of the analysis was to determine whether adherence to the Mediterranean diet could reduce the risk of developing dementia and Alzheimer’s disease. Based on the results, we provide recommendations for future research directions, particularly focusing on further exploring the relationship between the Mediterranean diet and neurodegenerative diseases.

**Table 2:** Eligibility criteria for study selection

Criteria	Description
Inclusion criteria	
Population (P)	Adult individuals diagnosed with dementia or Alzheimer’s disease, or those with cognitive decline
Exposure (E)	Assessment of adherence to the Mediterranean diet through validated tools, such as questionnaires
Comparison (C)	Not specified, as the focus is on exposure to the Mediterranean diet and its outcomes
Outcome (O)	Development of dementia, Alzheimer’s disease, or cognitive decline
Study design (S)	Observational studies, including cross-sectional, cohort, and case–control studies. Preference was given to studies with a longer follow-up period if multiple publications were based on the same population
Exclusion criteria	
Population	Studies that did not examine populations related to the Mediterranean diet
Non-human studies	Studies conducted on non-human subjects (e.g., animal experiments, in vitro studies)
Publication type	Studies that were not full-text publications (e.g., conference abstracts)
Language	Studies not published in English or Hungarian, with translations unavailable

### Statistical analysis to determine the overall effect

We conducted statistical analyses using the web-based tool available at MetaAnalysisOnline.com. To synthesize the data from the included studies, we employed a random-effects model to calculate the pooled hazard ratios (HRs) and the 95% confidence intervals (CIs). This model was chosen to account for the potential heterogeneity across studies, thereby allowing for greater generalizability of the findings. Forest plots were generated to visually represent both the individual study outcomes and the overall pooled effect. These plots provide a clear graphical summary of the effect estimates across studies, facilitating the comparison of study-level data and the identification of potential variations in results.

To quantify heterogeneity, we utilized the chi-square test (Cochran’s *Q*) alongside the *I*^*2*^ statistic. The chi-square test assesses whether the observed variability in effect sizes exceeds that expected by chance, while the *I*^2^ index quantifies the proportion of the total variability in the effect estimates that is due to between-study heterogeneity rather than sampling error.

### Identification of potential publication bias

We also evaluated potential publication bias by generating a funnel plot, which plots effect sizes against their standard errors to detect asymmetry, a sign of bias. To further assess the presence of publication bias, we conducted Egger’s test, a statistical method that examines the relationship between the effect estimates and their precision to detect significant deviations from symmetry.

### Assessing sample size robustness through trial sequential analysis

In addition to the primary metaanalysis, we performed a trial sequential analysis (TSA) to assess the robustness of the cumulative sample size and determine whether the evidence was sufficient to draw reliable conclusions. This approach enables us to determine whether the cumulative evidence had reached the threshold required for statistical significance or if further studies were needed to solidify the findings. TSA was conducted using the *metacoumbounds* package in Stata version 14.1. For this analysis, we assumed a relative risk reduction of 15%, with a two-sided *α* level of 5% and statistical power set at 80%. These parameters were chosen to estimate the required a priori information size (APIS), representing the minimum number of participants necessary to detect a statistically significant effect with adequate power.

### Subcohort analysis settings

The statistical analysis was conducted separately in each analyzed clinical setting, covering three main disease cohorts: cognitive impairment, dementia, and Alzheimer’s disease. In the analysis, we included cross-sectional, cohort, and case–control studies, all of which were incorporated based on the investigated condition into the meta-analysis.

## Results

**Fig. 1:**
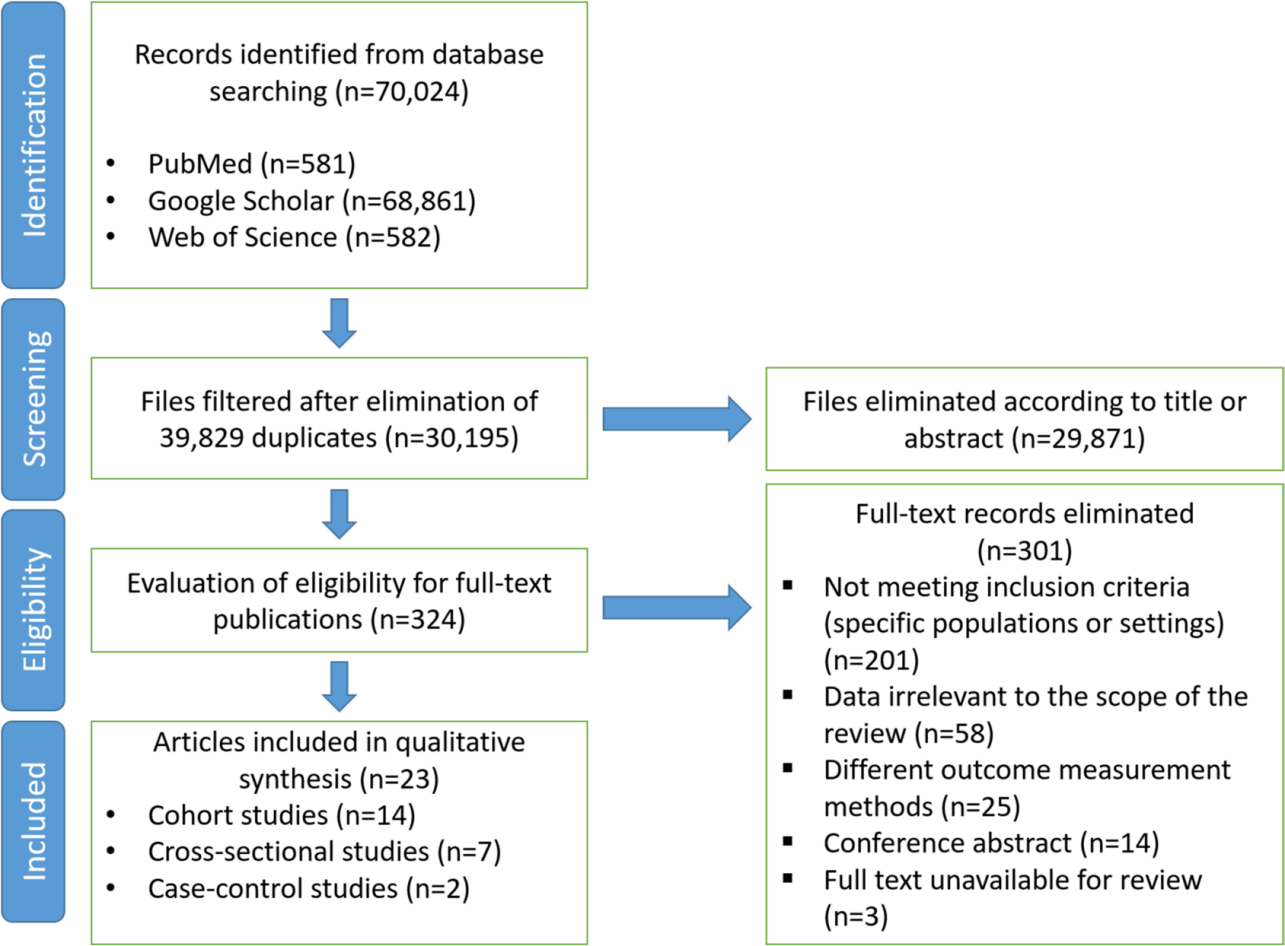
Flow diagram illustrating the article selection process

A total of 23 studies were included in the meta-analysis that met the inclusion criteria and provided analyzable data on the relationship between the Mediterranean diet and cognitive decline/mild cognitive impairment (MCI), dementia, and Alzheimer’s disease (Fig. 1). The summary of the results revealed several important correlations and findings.

### The relationship between the Mediterranean diet and cognitive decline

**Fig. 2:**
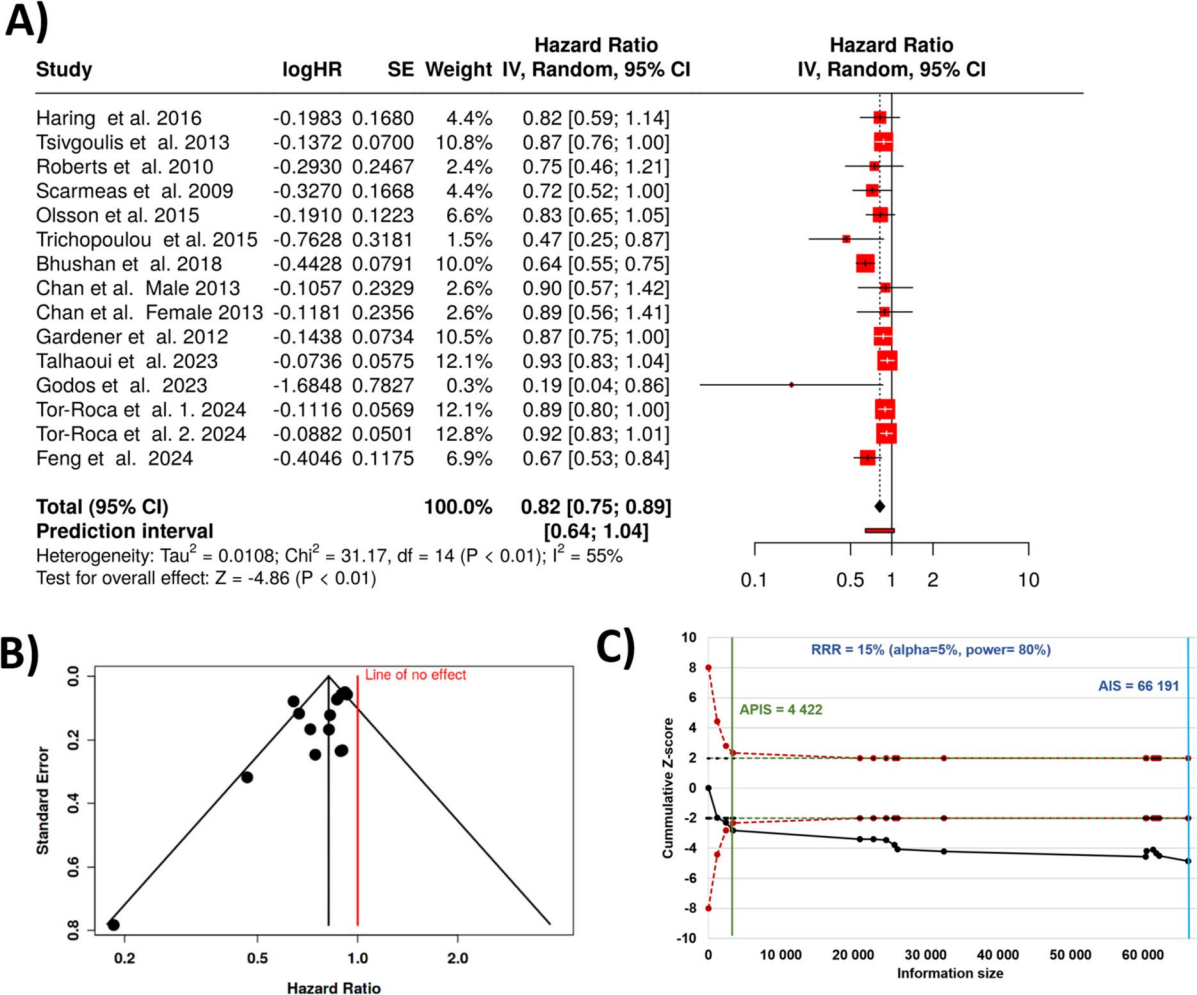
Results for all studies comparing Mediterranean diet and cognitive impairement. There is a highly significant reduction in cognitive impairement with a total HR of 0.82 (**A**). The funnel plot indicates a potential publication bias across the studies (**B**). The *Z*-score plot of all studies investigating the correlation indicates that no additional studies are needed to get a definitive conclusion (**C**). SE, standard error; CI, confidence interval; IV, inverse variance; APIS, a priori information size; AIS, actual information size; RRR, relative risk ratio

All together 13 studies analyzed examined the presence of cognitive decline as the prevalence of mild cognitive impairment (MCI) among individuals adhering to the Mediterranean diet [58–70]. The findings indicate that a closer adherence to the Mediterranean diet significantly slowed the rate of cognitive decline. Based on the analysis results using random effects model with inverse variance method to compare the hazard rate (HR), a statistical difference is present, and the summarized hazard rate (HR) is 0.82 with a 95% confidence interval of 0.75–0.89. The analysis for overall effect indicates a statistical significance with a *p* value below 0.05 (see Fig. 2A for the fores-plot showing all included stuies). The aggregated data showed that greater adherence to the Mediterranean diet reduced the rate of cognitive decline by approximately 18%, and participants who followed the Mediterranean diet maintained better cognitive function over time.

A significant heterogeneity was found (0.01), pointing at varying effects in scale and/or direction among the included studies. An *I* [2] value of designates that 55% of the inconsistency between trials stems from heterogeneity rather than random chance. In addition, the funnel plot indicates a potential publication bias. The Egger’s test supports the presence of funnel plot asymmetry (intercept: − 1.56; 95% CI − 2.79 to − 0.34; *t* − 2.503; *p*-value 0.026; Fig. 2B).

### The relationship between the Mediterranean diet and the risk of dementia

**Fig. 3:**
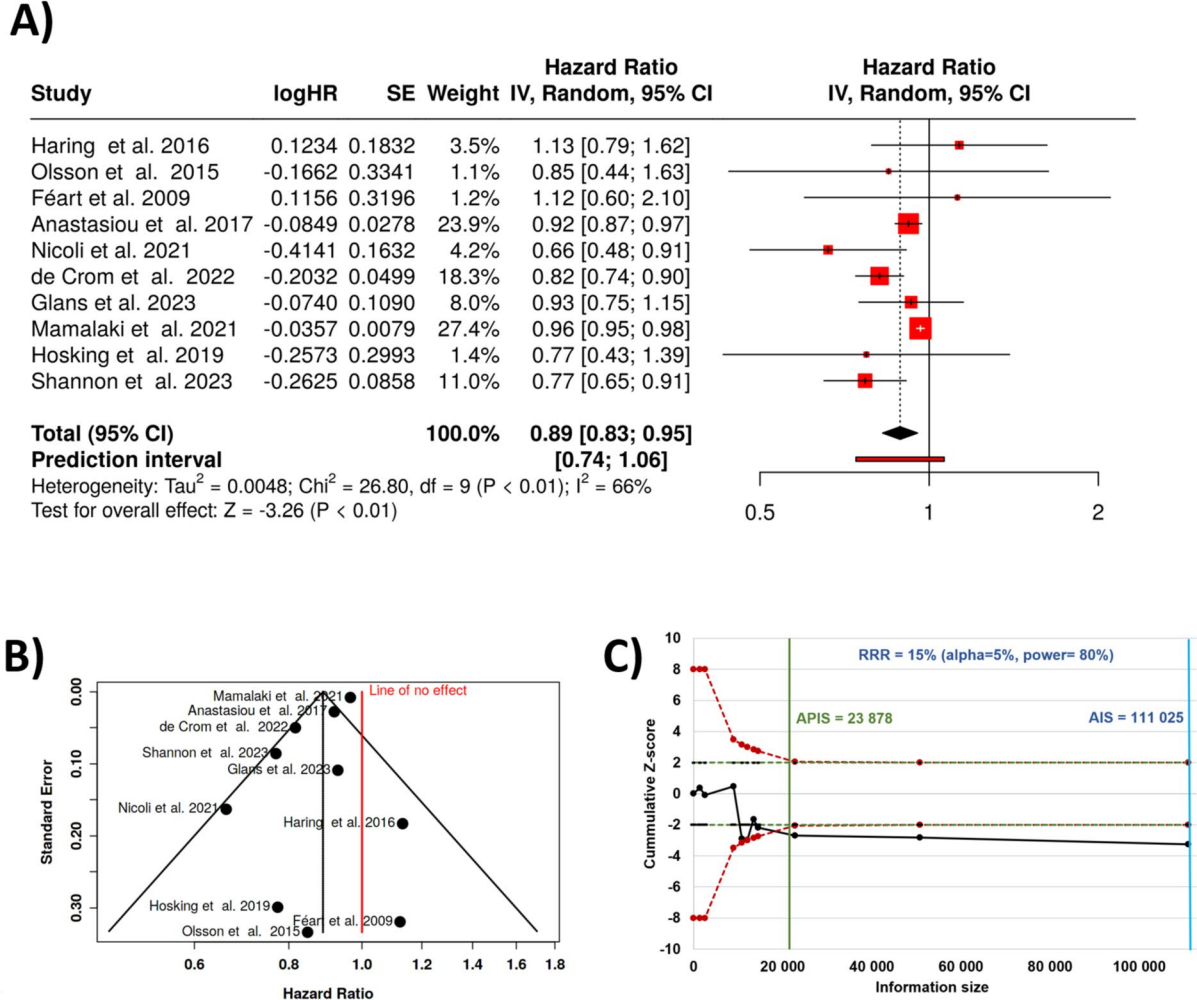
Results for all studies comparing Mediterranean diet and dementia. There is a significant reduction in the prevalence of dementia with a total HR of 0.89 (**A**). The funnel plot confirms the absence of a potential publication bias (**B**). The *Z*-score plot of trials analyzing the effects show that additional studies are not needed to get a definitive conclusion (**C**). SE, standard error; CI, confidence interval; IV, inverse variance; APIS, a priori information size; AIS, actual information size; RRR, relative risk ratio

Most of the analyzed studies found a significant inverse association between adherence to the Mediterranean diet and the risk of dementia. The aggregated results calculated from ten trials [58,62,71–78] using a random-effects model indicated that higher adherence to the Mediterranean diet reduced the risk of developing dementia by 11%. In particular, there is a statistical difference, and the summarized hazard rate (HR) is 0.89 with a 95% confidence interval of 0.83–0.95. The assessment for overall effect confirms a statistical significance with a *p* value below 0.05 (see Fig. 3A).

A significant heterogeneity was found (< 0.01), suggesting varying effects in scale and/or direction. An *I*^2^ value of indicates that 66% of the differences between the cohorts arises from heterogeneity rather than random chance (Fig. 3B). Meanwhile, the funnel plot does not indicate a potential publication bias and the Egger’s test does not support the presence of funnel plot asymmetry (intercept: − 1.11; 95% CI − 2.21 to − 0.02; *t* − 1.999; *p*-value 0.081).

### The relationship between the Mediterranean diet and the risk of Alzheimer’s disease

**Fig. 4:**
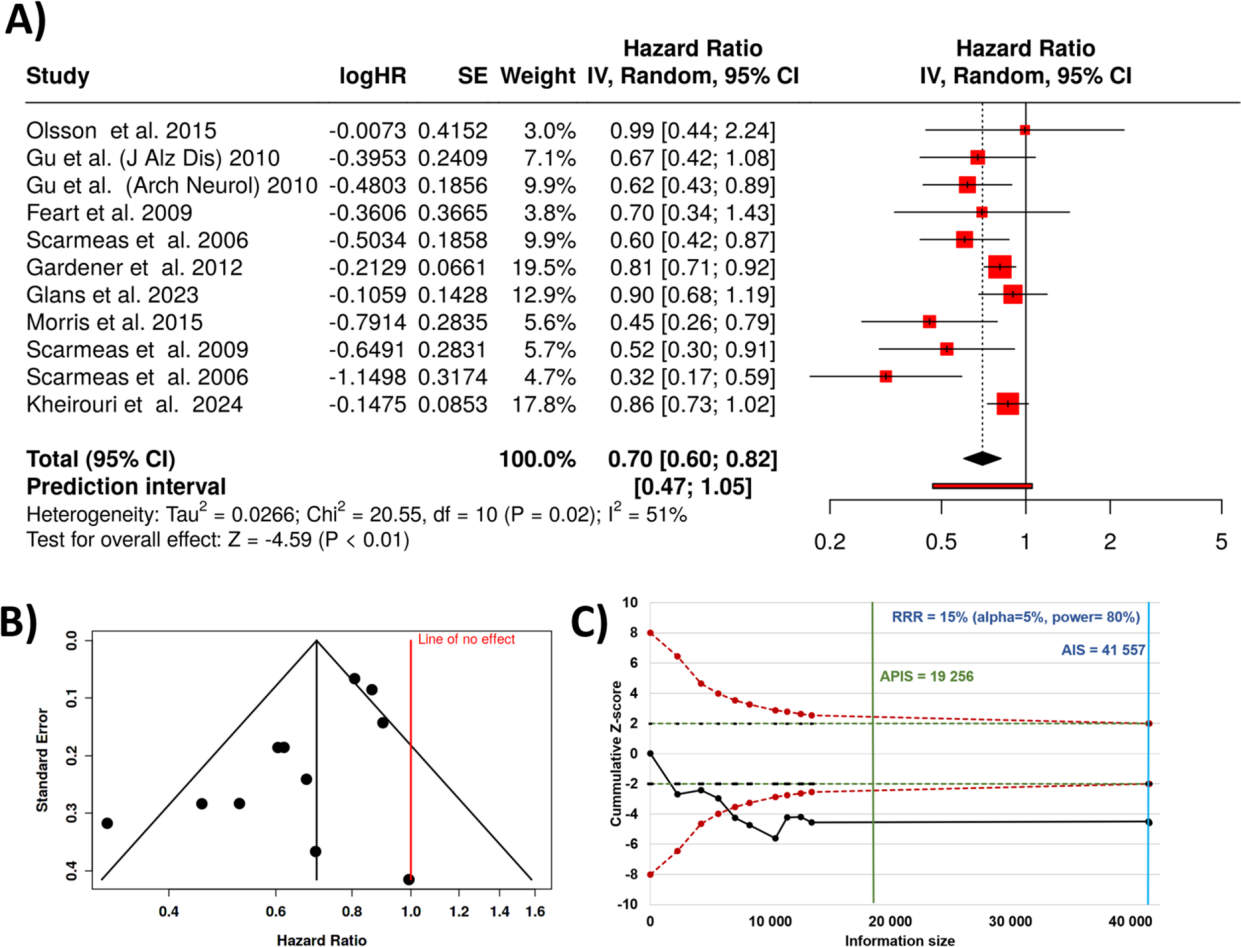
Results for all studies comparing Mediterranean diet and the incidence of Alzheimer’s disease. There is a 30% reduction in the incidence of Alzheimer’s disease (**A**). The funnel plot displays significant assymetry (**B**). The TSA analysis of studies investigating the correlation supports the sufficiency of the sample number to draw a final conclusion (**C**). SE, standard error; CI, confidence interval; IV, inverse variance; APIS, a priori information size; AIS, actual information size; RRR, relative risk ratio

In our meta-analysis, we also examined the impact of adherence to the Mediterranean diet on the risk of developing Alzheimer’s disease. The findings showed that the Mediterranean diet significantly reduced the risk of Alzheimer’s disease. A total of nine cohorts were investigated [62,66,71,75,79–83], and based on the calculations performed using random-effects model with inverse variance method to compare the hazard rate, there is a statistical difference, and the summarized hazard rate is 0.7 with a 95% confidence interval of 0.6–0.82. The analysis for overall effect points to a p value below 0.05. A significant heterogeneity was detected (*p* = 0.02), signifying variable effects in scale and/or direction. An *I*^2^ value of 51% marks the proportion of inconsistency among the cohorts arising from heterogeneity rather than random chance (Fig. 4A).

Thus, according to the random-effects model, the Mediterranean diet was associated with a robust 30% reduction in the likelihood of developing Alzheimer’s disease. This result suggests that the Mediterranean diet may have a protective effect against Alzheimer’s disease, particularly with long-term adherence. Newertheless, the funnel plot indicates a potential publication bias and the Egger’s test supports the presence of funnel plot asymmetry (intercept: − 1.63, 95% CI − 2.84 to − 0.41; *t* − 2.626; *p*-value 0.028; see Fig. 4B).

### Trial sequential analysis

We performed TSA analysis in each of the three settings investigated. To aid in the interpretation of the TSA results, we generated *Z*-score plots, which display the relationship between the cumulative sample size, study duration, and cumulative *Z*-scores over time. The resultsing plots for cognitivie impariemen, dementia, and Alzheimer’s disease are provided in Fig. 2C, Fig. 3C, and Fig. 4C, respectively. In all three settings, the actual cumulative sampel number was much higher, than the original APIS. Thus, by applying TSA, we were able to account for the risks of random errors and we determined that the existing data is sufficient and no additional research is needed in order to avoid premature or inconclusive results.

## Discussion

The findings from this meta-analysis provide robust evidence supporting the protective role of the Mediterranean diet in reducing the risk of cognitive decline, dementia, and AD. The consistent associations observed across a range of studies highlight the potential of the Mediterranean diet as an effective non-pharmacological, lifestyle intervention for promotion of healthy cognitive and brain aging, mitigating the progression of both VCID and AD. These results are especially relevant given the increasing burden of age-related cognitive impairment and dementia in aging populations worldwide.

Both VCID and AD are age-related diseases, and their pathogenesis is closely linked to cellular and molecular mechanisms of aging [9,10,84–86]. It is logical to assume that the Mediterranean diet interferes with these aging processes [87–90], contributing to its protective effects against both conditions. This assumption is supported by the observation that the Mediterranean diet also protects against other age-related diseases, such as cardiovascular diseases [91], stroke [28,92–95], cancer [96–98], sarcopenia [99,100], and age-related macular degeneration (AMD) [101–104].

Components of the Mediterranean diet [105–109] likely exert their protective effects through a combination of vasoprotective and neuroprotective mechanisms, delivering anti-aging benefits to both vascular cells and cells of the central nervous system. A key feature of the Mediterranean diet is its richness in anti-inflammatory nutrients, such as omega-3 fatty acids, which are abundant in fish and nuts [57,110,111]. Additionally, plant-based foods like vegetables, fruits, and whole grains are rich in flavonoids and other bioactive compounds with powerful antioxidant and anti-inflammatory properties [57,112]. Extra virgin olive oil, a cornerstone of the Mediterranean diet, contributes to brain health due to its high content of monounsaturated fats and polyphenols [57,72,78,90,113]. The regular consumption of fruits and vegetables in this diet has been well-documented for its protective effects against cardiovascular diseases and cognitive decline. These foods are particularly rich in fiber, potassium, flavonoids, and carotenoids, all of which play a role in enhancing cardiovascular and cognitive health. Red wine, another notable component of the Mediterranean diet, is rich in polyphenols—particularly resveratrol—which may offer additional multifaceted health benefits.

One of the key protective mechanisms of the Mediterranean diet lies in its ability to counteract the cellular and molecular processes that drive aging, such as mitochondrial dysfunction [87,90], oxidative stress, and chronic inflammation [114]—all of which are major contributors to both VCID and AD [79]. Several components of the diet, including polyphenols, omega-3 fatty acids, and monounsaturated fats, are known to modulate these aging-related mechanisms, thereby supporting vascular and cognitive health.

Polyphenols (abundant in olive oil, fruits, vegetables, and red wine), including resveratrol, have strong antioxidant and anti-inflammatory properties. They scavenge free radicals, activate endogenous antioxidative defense mechanisms, such as Nrf2-regulated antioxidative responses and induce master regulators of pro-survival cellular programs such as SIRT1-mediated cellular stress resilience pathways [115,116], mitigate both mitochondrial and NADPH oxidase-dependent ROS production, reduce oxidative damage, improve mitochondrial health [115], and improve endothelial function by enhancing nitric oxide bioavailability [117,118], which is essential for maintaining vascular health. Polyphenols also inhibit the expression of pro-inflammatory cytokines and reduce the activation of inflammatory pathways, such as NF-κB, which are implicated in both microvascular dysfunction and neuroinflammation.

Omega-3 fatty acids, found in fish and nuts, play a critical role in neuroprotection by promoting neuronal cell function [119], reducing neuroinflammation [120], and enhancing synaptic plasticity [121,122]. These fatty acids also regulate cerebral blood flow [123,124] and lower the risk of atherosclerosis by reducing triglycerides, blood pressure, and inflammation [125,126]—factors that are vital for both cerebrovascular and cognitive health. Recent meta-analyses demonstrated that fish consumption is associated with a lower risk of cognitive impairment and dementia [127,128]. The neurocognitive protective effects of fish consumption also associates with a reduced risk of cardiovascular disease mortality [129]. Nuts, another essential component of the Mediterranean diet, are not only rich in phytochemicals and unsaturated fatty acids but are also a good source of folate, vitamin B6, and niacin [130]. Increased nut consumption has been associated with improved cognitive performance, further supporting their role in promoting brain health [131–133].

Monounsaturated fats, primarily sourced from olive oil, contribute to improved lipid profiles by lowering low-density lipoprotein (LDL) cholesterol and raising high-density lipoprotein (HDL) cholesterol [134–141]. These favorable changes help protect against vascular damage and enhance endothelial function, both of which are crucial for reducing the risk of microvascular pathologies that contribute to dementia. Data from the Three Cities Study [142], along with other clinical, translational, and preclinical research [143–148], have demonstrated the protective effects of olive oil against cognitive decline and neurodegeneration [149–151], further supporting its role in maintaining brain health.

In the pathophysiological processes of AD, amyloid-beta (Aβ) deposition and abnormal tau protein phosphorylation are key contributors to neuronal damage and cognitive decline. Aβ plaques form when amyloid precursor protein (APP) is improperly cleaved, leading to the accumulation of insoluble Aβ peptides in the brain, which disrupts synaptic function and triggers neuroinflammation. Similarly, hyperphosphorylated tau protein forms neurofibrillary tangles that interfere with intracellular transport and contribute to neuronal dysfunction. Numerous studies have shown that adherence to the Mediterranean diet can help reduce Aβ levels in the brain [152–154], potentially slowing the development and progression of AD [66,79–81]. Clinical and preclinical studies suggest that components of the Mediterranean diet can interfere with the molecular mechanisms underlying amyloid and tau pathologies [152–154]. For example, oleocanthal, a naturally occurring compound found in extra virgin olive oil, has garnered significant attention for its neuroprotective properties. Research has demonstrated that oleocanthal inhibits the formation and aggregation of Aβ oligomers, which are highly toxic to neurons [155–157]. By preventing the buildup of these oligomers, oleocanthal reduces amyloid plaque formation, helping to protect neuronal networks and preserve cognitive function [155–157]. Beyond oleocanthal, other bioactive components of the Mediterranean diet may also contribute to these protective effects. Polyphenols, such as resveratrol and flavonoids, have been shown to reduce oxidative stress and inflammation, which are closely linked to Aβ and tau pathologies [158]. These compounds modulate signaling pathways involved in amyloid clearance, reduce the production of pro-inflammatory cytokines, and promote autophagic processes that help remove misfolded proteins, further supporting the Mediterranean diet’s role in slowing AD progression [158]. The preclinical and clinical findings on the impact of omega-3 fatty acids mitigating the effects of Aβ and tau in the brain are controversial [159–161]. Studies have shown that these fatty acids can reduce neuroinflammation [120,162] and enhance synaptic plasticity [122]. Taken together, the Mediterranean diet, through its rich content of neuroprotective bioactive nutrients may provide a multifaceted approach to combatting the key pathological features of AD.

The Mediterranean diet has also been shown to reduce several vascular risk factors common to both VCID and AD, including hypertension [163,164], diabetes [165], obesity [166], and hyperlipidemia [114]. These risk factors contribute to cerebromicrovascular pathologies [167], such as microvascular rarefaction, microhemorrhages, and BBB disruption, which are critical in the genesis of ischemic neuronal damage and neuroinflammation, thereby promoting the development of dementia. In addition to the direct effects of the Mediterranean diet, its benefits may be enhanced by other lifestyle factors commonly associated with the Mediterranean way of life, including regular physical activity, strong social connections, and a slower pace of life [168]. These factors can further support cardiovascular health and mental well-being, creating a comprehensive approach to healthy aging.

The findings of this meta-analysis on the protective effects of the Mediterranean diet against cognitive decline and dementia carry significant public health relevance for regions where the incidence of cognitive impairment and dementia is high, and dietary habits differ markedly from the traditional Mediterranean lifestyle [1]. Hungary is a prime example. The country faces alarming statistics regarding unhealthy cognitive aging, with dementia prevalence rates steadily increasing due to both an aging population and widespread unhealthy lifestyle habits [169]. Cognitive decline is a leading cause of disability in older adults in Hungary. Additionally, the country ranks among the highest in Europe for cardiovascular-related mortality, which is closely linked to the risk of VCID [170–173]. As the population continues to age, Hungary is facing a growing burden of cognitive decline and dementia, placing substantial strain on the healthcare system. The increasing incidence of dementia in Hungary reflects broader public health challenges related to unhealthy aging, which is largely driven by poor dietary habits, physical inactivity, and other unfavorable lifestyle factors. Like many Central and Eastern European countries, Hungary has a high prevalence of risk factors such as hypertension, obesity, diabetes, and hyperlipidemia, all of which exacerbate the progression of both AD and VCID. These conditions are closely tied to dietary patterns that are low in fruits, vegetables, whole grains, and healthy fats [174]—key components of the Mediterranean diet. The findings of this meta-analysis provide a strong foundation for embedding the Mediterranean diet into public health initiatives in Hungary, positioning it as an effective, non-pharmacological intervention to reduce the risk of cognitive decline, dementia, and cardiovascular and cerebrovascular diseases. This approach aligns well with Hungary’s existing public health research programs, such as the Semmelweis Study [175] and the Semmelweis-EUniWell Workplace Health Promotion Program, which aim to address the causes of unhealthy aging and implement targeted interventions. By incorporating the Mediterranean diet, these programs could amplify their impact, addressing dietary gaps and enhancing the health and well-being of aging populations more effectively.

Our meta-analysis has provided valuable insights into the potential protective effects of the Mediterranean diet against cognitive decline and dementia, but several limitations must be acknowledged. First, significant heterogeneity among the included studies complicated the clear interpretation of the results. This heterogeneity likely arises from differences in study design, population characteristics, dietary adherence assessment methods, and variations in the composition of the Mediterranean diet across different regions, as the diet is not a monolithic entity but rather a collection of dietary patterns that vary significantly. Additionally, other Mediterranean lifestyle factors, such as physical activity and social engagement, may have confounded the results, making it difficult to isolate the specific effects of the diet itself. While it is possible to attempt to address these issues through subgroup analyses, methodological differences and varying population characteristics among the studies could still influence the results. Second, most of the studies included in this meta-analysis were observational in nature, which limits the ability to draw definitive causal inferences. Although observational studies provide valuable information, they are inherently limited by potential confounding variables and biases. Therefore, randomized controlled trials are needed to strengthen the causal basis of the relationship between the Mediterranean diet and cognitive health. Third, the possibility of publication bias must be considered. Studies with positive results are more likely to be published, which could skew the findings of the meta-analysis. Although funnel plots and statistical tests did not indicate significant publication bias, it remains possible that some studies with null or negative results were not published. To address this limitation, future research should prioritize the publication of null or negative results and emphasize the importance of preregistering study protocols to ensure transparency and reduce selective reporting. Finally, the majority of studies included in this meta-analysis were conducted in Mediterranean or Western populations. This raises questions about the generalizability of our findings to ethnically diverse or non-Mediterranean populations, where dietary habits, genetic predispositions, and environmental factors may differ significantly. Future research should explore the impact of adherence to Mediterranean dietary patterns in non-Mediterranean regions, particularly in regions where the incidence of cognitive decline and dementia is high and dietary habits differ significantly from the traditional Mediterranean lifestyle. This would provide a more comprehensive understanding of the diet’s broader applicability in preventing neurodegenerative diseases. Tailored interventions considering local dietary preferences and cultural practices are necessary to optimize the diet’s potential benefits globally.

In summary, our meta-analysis confirms that adherence to the Mediterranean diet significantly reduces the risk of dementia and Alzheimer’s disease. This diet, abundant in antioxidants, anti-inflammatory nutrients, and healthy fats, plays a crucial role in preserving cognitive function and preventing neurodegenerative diseases. Based on our findings, we advocate for the inclusion of the Mediterranean diet in dietary strategies targeting dementia and Alzheimer’s prevention, especially in high-risk populations. Further research is needed to identify the specific components of the Mediterranean diet that most effectively prevent cognitive decline and to explore the duration and extent of its neuroprotective benefits. Future randomized controlled trials are essential to validate the effectiveness of dietary interventions and to explore how the Mediterranean diet may be optimally combined with other preventive measures to further mitigate the risk of dementia and Alzheimer’s disease.
